# Recove® burn ointment for managing acute radiodermatitis in patients with breast cancer: A double blind randomized controlled trial

**DOI:** 10.22088/cjim.13.2.

**Published:** 2022

**Authors:** Nargeuss Abbaszade Marzbali, Ebrahim Zabihi, Alexis Vallard, Nicolas Magne, Mohammad Moslemi, Dariush Moslemi

**Affiliations:** 1 Cellular and Molecular Biology Research Center, Babol University of Medical Sciences, Babol, Iran; 2 Student Research Committee, Deputy of Research and Technology, Babol University of Medical Sciences, Babol, Iran; 3 Department of Physiology and Pharmacology, Babol University of Medical Sciences, Babol, Iran; 4 Department of Radiotherapy, Lucien Neuwirth Cancer Institute, Saint-Priest en Jarez, France; 5 Dentistry student, Babol University of Medical Sciences, Babol, Iran; 6 Department of Radio Oncology, Babol University of Medical Sciences, Babol, Iran

**Keywords:** Breast cancer, Radiotherapy, Radiodermatitis, Sesame oil, Camphor, Zinc oxide

## Abstract

**Background:**

Radiodermatitis is the most common complication of radiotherapy. There is no gold standard for managing the radiodermatitis. This study aimed to evaluate the effect of topical Recove^®^ burn ointment; basically compounded of sesame oil, camphor, and zinc oxide; in preventing acute radiodermatitis.

**Methods:**

This double blind RCT (IRCT No.: 201204047136N2) was performed on 71 patients that referred for radiotherapy after mastectomy to Shahid Rajaee Hospital (Babolsar-Iran) during 2013-2017. Patients were allocated into 2 groups; 34 in control group and 37 in Recove^®^ group. Patients applied the ointment 2 times a day, before every radiation therapy session for 5 weeks. The radiation oncologist assessed the severity of dermatitis weekly for 5 weeks and graded it from 0 to 4 according to the RTOG criteria.

**Results:**

Baseline characteristics including age, and BMI had no significant difference between groups. The Recover group patients experienced significantly less severe dermatitis compared to the controls (p<0.001). None of the patients in Recove® group encountered more than grade 2 of RTOG criteria, however, in the control group, 4 (12.9%) patients experienced grade 3 of RTOG and 3 (9.7%) patients developed grade 4 of RTOG at the end of the 5^th^ week.

**Conclusion:**

Our results indicate that Recove^® ^ointment significantly reduces the severity of acute radiodermatitis.

Radiotherapy has evolved as a powerful tool for tumor control or as a treatment(1). But like any other treatments, radiotherapy has an adverse effect. One of the most important side effects of this procedure is radiation-induced skin toxicity which is known as radio dermatitis. It affects up to 74-100 % of breast cancer patients where 85 % of these patients develop a moderate-to-severe dermatitis. Skin dryness, warmth, and burning sensation followed by erythema, desquamation, ulceration even necrosis which are some of mostly reported signs and symptoms .Furthermore several studies indicate that acute skin reactions are a relevant risk factor for the occurrence of late skin toxicities (2). Morphologic and functional changes that occur in noncancerous tissue are a direct result of ionizing radiation (3). These changes include: pain, discomfort, irritation, itching, and burning. Development of radio dermatitis may begin immediately after 2-3 weeks of radiotherapy initiation, and may persist up to 4 weeks after treatment ends and interrupts complete treatment (4). There are some tools to evaluate the severity of radiodermatitis. One of the most feasible tools is published by the radiation therapy oncology group which is called RTOG scale (5).

In this study, we use this tool to measure radiodermatisis severity after radiotherapy for breast cancer. As radiodermatitis is the most common side effect of radiation therapy and a variety of topical agents (including: aqueous cream, corticosteroid, Biafin cream, Aloe Vera, antibacterial agents, anti-oxidant, Sucralfate) and systemic treatments (such as: amifostine, oral hydrolytic enzymes, pentoxifylline and zinc supplement) are used to ameliorate the side effect, but there are few publications to provide required shreds of evidence to support their wide use (6-9). 

There are different compounds in traditional and modern medicine used for wound healing such as sesame oil, camphor, and topical zinc. Sesame oil, delivered from the plant sesamum, in traditional medicine is used to relieve pain in people with joint pain, toothache, premenstrual syndrome, scrapes and cuts. The anti-inflammatory properties of sesame oil are proven(10). Camphor is isolated from the wood of the camphor laurel tree which is used commonly for skin antipruritic, analgesic and counterirritant properties(11). Topical zinc is widely used in wound treatment(12). Inhibition of human gelatinase activity by zinc oxide is a possible mechanism to enhance wound healing(13). There is a report of antibacterial effect of zinc oxide on Pseudomonas aeruginosa, Staphylococcus Auereus and Streptococcus Pyogenes which plays an important role in wound treatment (14). 

Recove^® ^is an Iranian burn ointment that contains sesame oil, zinc oxide and camphor. This ointment is now used to modify inflammation due to laser therapy, burns and sunburn. There is no study to support the effect of this product for the prevention or treatment of radio dermatitis. We hypothesized that Recover can be effective for the prevention and treatment of post-radiotherapy radiodermatitis. This study aimed to assess the efficacy of Recove^®^ burn ointment for radiodermatitis in patients with breast cancer receiving radiation therapy.

## Methods

**Patients and Settins:** This randomized controlled trial, was conducted in Shahid Rajaee Radiation Therapy Hospital of Babolsar City of Mazandaran province in the North of Iran from 2013 to 2017 as two-arm superiority trial with 1:1 allocation ratio. The study was first registered in Babol University of Medical Sciences Ethics Committee with and then in Iranian trial registry database (IRCT) with registration number 201204047136N2. Participants were selected from consecutive patients who had a definite diagnosis of breast cancer referred for radiation therapy after modified radical mastectomy and chemotherapy. There was at least 4 weeks interval between the end of chemotherapy and the start of radiotherapy. Patients with a history of diabetes mellitus, prior radiotherapy to the chest wall and any collagen vascular disease or any dermatologic conditions which could misinterpret our physical examination were excluded from the study. 80 patients were assessed for eligibility. 4 patients had one of the exclusion criteria.

**Figure 1:**
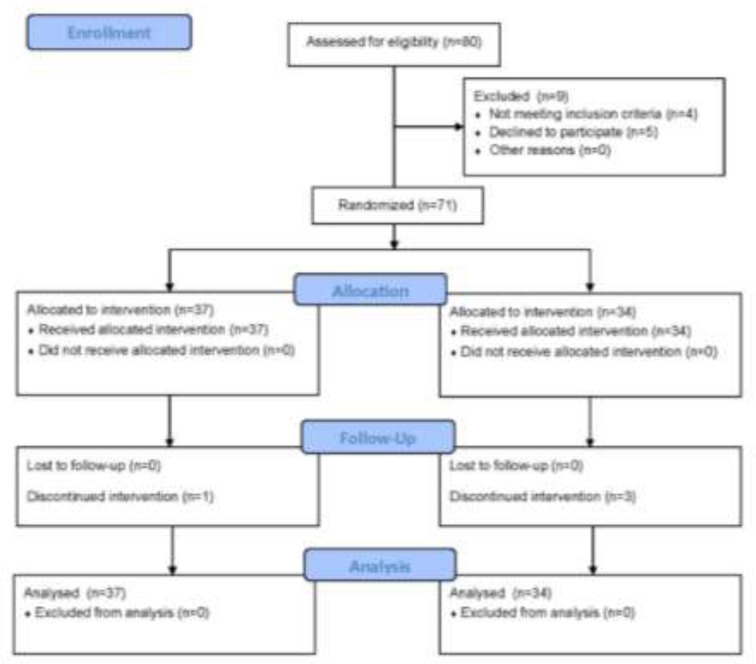
CONSORT flow diagram of our RCT indicate the number of enrolled, allocated, and followed up patients

The remaining 76 patients were randomly allocated into two 38 patients groups. Informed written consent has been taken from the patients. 1 patient in the intervention group and 4 patients in the control group did not accept to sign the informed consent form. Finally 71 (37 in intervention group and 34 in control group) patients participated in the trial. The intervention group received Recove^® ^ointment and the control group received petrolatum ointment as placebo. The patients received 50 GY of radiation to the chest wall by external beam electrons (6 MV) weekly with an applied dosage of single fraction of 200 cGY per day, five times a week for five weeks. Chest wall field arrangement included the area between the midsternal line medially, midaxillary line laterally, 2cm below the contra lateral inframammary fold inferiorly, and supraclavicular-axillary field superiorly. Patients were followed-up four weeks after the intervention and physical examination for skin lesions done every week by a radio oncologist. In the third week, 1 patient in intervention group and 1 patient in the control group were lost to follow-up because of patient’s noncompliance. Also, in the fifth week, 2 other patients in control group were lost to follow-up because of the same reason. The procedure of patient eligibility, random allocation, and follow-up periods was briefly shown in figure 1 based on CONSORT flowchart.

**Blinding and clinical assessment:** This trial was double-blinded. Recove^® ^ointment (manufactured by Tousan Daru Co., Tehran, Iran) was arranged for intervention group. Petrolatum ointment as a placebo provided for the control group (manufactured by Tousan Daru Co., Tehran, Iran) exactly in same tubes looks like the Recove^® ^ointment tubes. To double blind the study, 80 randomly coded ointment tubes were filled either by Recove® or petrolatum (an inert excipient used for the ointment base) by the pharmacist and the ointment tubes codes were kept safe until the end of study. Neither the physician nor the patients were aware of the administered ointment ingredients until the codes were broken down. The pharmacist disclosed the codes after data collection was completed. Before every radiation therapy session, the patients washed the radiotherapy area by a neutral soap and water. Every weekend during radiotherapy and till 1 week after the end of radiotherapy, skin injury was scored according to the radiation therapy oncology group (RTOG) criteria by radiation oncologist- grade 0: no change over baseline; grade 1: follicular, faint or dull erythema, epilation, dry desquamation, decreased sweating; grade 2: tender or bright erythema, patchy moist desquamated, moderate erythema; grade 3: confluent, moist desquamation other skin folds, pitting edema; and grade 4: ulceration, hemorrhage, necrosis.

**Statistical analysis:** The codes were disclosed after completion of data collection. Data were analyzed using SPSS software Version 12 (Chicago). Baseline characteristics were compared between the two groups using independent sample t-test and chi-square test. The outcome used for analysis was the frequency and severity of skin injuries between two groups with chi-square tests. Quantitative assessment of radiodermatitis severity in 5 weeks was analyzed using repeated measures test. A p-value less than 0.05 was considered significant.

## Results

After the exclusion of patients who did not meet the eligibility criteria and who denied to participate in the study, finally the data of 71 patients were enrolled in analysis. 37 patients were allocated in the intervention group and 34 patients in control group (fig. 1). The mean age of all patients was 50.26±11.65 and all of them were women. The baseline characteristics of patients include: age, weight, height, BMI, and pathology had no statistically significant difference between the two groups (table 1). 

**Table 1:** Baseline characteristics of patients in control and intervention group

**group** **Characteristics**	**Control**	**Intervention**	**P value**
Agemean ± SD	49.8±9.83	50.68±13.4	0.844
BMI mean ± SD	29.87±5.50	29.85±4.83	0.960
Pathology (IDC^*^) n (%)	30 (81.1)	29 (85.3)	0.636

* IDC= Infiltrating Ductal Carcinoma

All skin injuries examination was performed over 5 weeks. Patients in intervention group experienced dermatitis no greater than grade 2 while the patients in control group showed grade 3 or grade 4 dermatitis in follow-up period. In week to week analysis, no patients in both groups developed acute dermatitis at the end of the first week. But in the second week, frequency of patients with grade 1 dermatitis was significantly more in control group than the Recove^®^ group. In the third week, most of the control group (48.5%) patients had grade 1 dermatitis, in contrast to most of Recove treated (72.9%) patients with no dermatitis. This difference in the third week between two groups was statistically significant. In the fourth week like the third week, most of the control group patients (51.1%) suffer from grade 1 dermatitis but most of Recove treated patients (48.6%) had no dermatitis. And finally in the fifth week, most of patients in the control group (56.1%) suffer from grade 2 dermatitis but most patients in Recove treated group (47.2%) suffer from grade 1 dermatitis and this difference was significant. Details of frequency of each RTOG grade dermatitis in every week in both groups were shown in table 2. 

**Figure 2:**
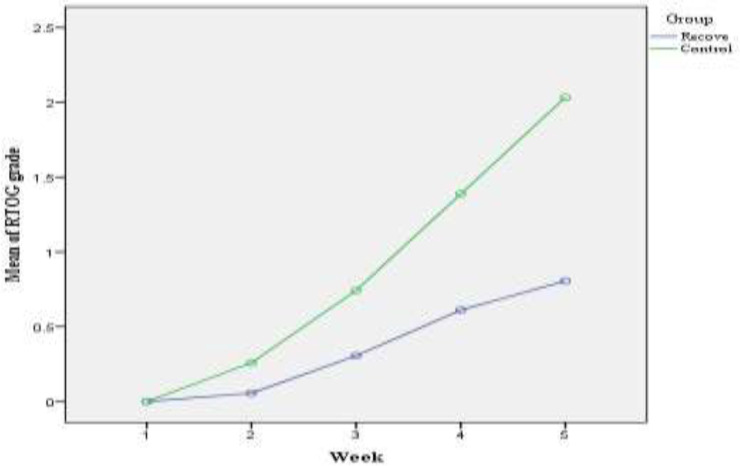
Repeated measures analysis showed that Recove treated patients had lower mean of RTOG grade score in comparison to control group (P<0.001)

Quantitative assessment of dermatitis grade in both groups with general linear model analysis with repeated measures indicated that the mean score of dermatitis grade in five weeks in the intervention group is significantly lower than the control group (figure 2).

**Table 2:** Frequency of RTOG grades dermatitis in patients of control and intervention group in study period

** Grade** **Week**		**Grade0** n (%)	**Grade1** n (%)	**Grade2** n (%)	**Grade3** n (%)	**Grade4** n (%)	**P-value**
1^st^ wk	Recove^®^	37(100)	0	0	0	0	­
	Control	34(100)	0	0	0	0	
2^nd^ wk	Recove^®^	33(94.6)	2(5.4)	0	0	0	0.015
	Control	25(73.5)	9(29.5)	0	0	0	
3^rd^ wk	Recove^®^	26(72.2)	9(25)	1(2.8)	0	0	0.014
	Control	13(39.4)	16(48.5)	4(12.1)	0	0	
4^th^ wk	Recove^®^	18(48.6)	14(40.54)	4(10.81)	0	0	0.001
	Control	4(12.1)	17(51.1)	8(24.2)	4(12.1)	0	
5^th^ wk	Recove^®^	13(36.1)	17(47.26)	6(16.7)	0	0	<0.001
	Control	1(3.2)	7(26.22)	16(56.1)	4(12.9)	3(9.7)	

## Discussion

In the present study, we have shown that the topical application of Recove^®^ ointment could have a protective effect on skin reactions during and following breast irradiation. Recove^® ^burn ointment is an Iranian topical agent used primarily as a burn cream but there was no study to assess the effect of Recove^® ^in the prevention or managing of radiodermatitis. Despite advances in radiologic technology and supportive care, radiodermatitis is not well managed yet(15). The use of the different topical agents and systemic treatments have contradictory results and few of them demonstrated an effective prevention of skin injury(16). The result of our study indicated that the patients who received Recove^® ^encountered less severe skin damage. Radiotherapy is a critical component in the treatment of breast cancer but it is often associated with bothersome skin reactions that has an impact on pain and quality of life in this patient, and if severe, may necessitate changes to the patient’s radiation schedule or treatment interruption(17, 18).

One of the components of Recove^® ^ointment is zinc oxide. The effect of this element on the management of wound healing and skin dermatitis was evaluated in some studies (12, 19, 20). Gorodetsky and et al. in 1999 in a pilot study evaluated efficacy of a self-manufactured zinc oxide cream on radiation-induced dermatitis of patients with breast cancer. They found that zinc oxide cream had protective effect on radiodermatitis (21). In another study by Lin and et al. in 2006 worked on the effect of oral supplement of zinc in the prevention of radiodermatitis in patients with head and neck cancers after radiotherapy (22). They showed that zinc supplement would postpone development of dermatitis in comparison to control group. These two studies argue our findings with Recove^®^. We did not find any study which counter argue our study both in human and animal models of radiodermatitis. There are studies showing that zinc and its derivations had antibiotic activity specially against Pseudomonas aeruginosa (14) and also studies which showed that zinc can inhibit matrix metalloproteinase 2 and 9 (13). These two mechanisms may explain the anti-inflammatory and wound healing effect of zinc which is a component of Recove^® ^ointment.

Another component of Recove^®^ ointment is sesame oil. Anti-inflammatory and anti-fungal effect of sesame oil was proven in some in vivo and in vitro studies (23-25), but there were not any clinical studies on this issue. Otherwise there are some studies indicating that sesame oil would cause allergic reactions either in oral consumption (26) or contact with skin (27). Although this evidence may counter argue our findings, but we found that patients in Recove^®^ ointment-treated group underwent scaling without necrosis in sub-scaled area. With regard to our results that Recove^®^ can prevent radiodermatitis, and according to the clinical evidence of allergic potential in sesame oil, it can be considered that sesame oil of Recove^®^ can cause scaling of dead tissues and allow the skin to regenerate itself.

In traditional medicine, camphor is used as an antiseptic, analgesic and skin antipruritic agent (11). We could not find any clinical studies on camphor use for radiodermatitis or dermatitis. But according to the present study, the severity of skin injury with the Recove^® ^was significantly lower than control group and they did not develop toxicity more than grade 2 of RTOG. These results may indicate that camphor has anti-inflammatory effect and future studies will clarify the effect of camphor on radiodermatitis.

Drugs other than Recove^® ^components were used in the treatment of radiodermatitis. Vavassis et al. used the silver leaf nylon dressing for the treatment of RTOG grade 2 dermatitis in patients with head and neck region cancer but they found no improvement (28). Hemati et al.’s study compared the effect of silver sulfadiazine (SSD) cream on the prevention of radiodermatitis in patients with breast cancer and showed that the incidence of RTOG grade 3 skin injury was 3.9% in the 5^th^ week and 21.5% in the first week after the end of radiotherapy (3), however, in our study we had no patients that encountered the RTOG grade more than 2. Fiets et al. in 2001 conducted a randomized prospective trial of 74 patients with breast cancer receiving radiation. They were randomized to Biafin or no treatment. The results supported no improvement in Biafin group compared to patients with no treatment (29). In contrast Pommier et al. demonstrated Biafin (Trolamin) cream can effectively reduce the acute radiation- induced dermatitis (30). In another study, no significant advantages of Pentoxifylline prophylaxis on the development of acute skin reactions were reported in Aygenc et al.’s article (31). It seems that Recove^® ^may be a more effective ointment in comparison to other drugs used for radiodematitis treatment but further studies are needed to support this evidence in a stronger way.

There are publications which indicate different findings on how to manage radiodermatitis, but no gold standard exists for the prevention or treatment of radiation-induced dermatitis. In this study, for the first time, we evaluated the effect of Iranian ointment Recove^® ^ on radiodermatitis. We performed this study only in one center of radiotherapy and we have limited available patients. From the cultural point of view, there was a tendency among the patients not to participate in medical trials in general. Since written consent was necessary in this study, some patients denied undergoing even primary assessment. Multicenter trials with large number of sample size would shine the effect of this ointment on radiation-induced dermatitis better. In this study, we assessed the effect of Recove^® ^on chest wall dermatitis in breast cancer patients. Study on other region of body in other cancers also can determine the preventive and curative effect of Recove on post radiotherapy dermatitis.

In conclusion, our study showed that Recove^®^ointment had significant curative effect on radiation-induced dermatitis of the chest wall of breast cancer patients. We suggest radio oncologists to use this ointment for the prevention and treatment of radio dermatitis in patients with breast cancer. But more researches are needed to support the evidence that we found and compared the beneficial effects of Recove^® ^with other agents.
